# A robust gene signature for the prediction of early relapse in stage I–III colon cancer

**DOI:** 10.1002/1878-0261.12175

**Published:** 2018-02-16

**Authors:** Weixing Dai, Yaqi Li, Shaobo Mo, Yang Feng, Long Zhang, Ye Xu, Qingguo Li, Guoxiang Cai

**Affiliations:** ^1^ Department of Colorectal Surgery Fudan University Shanghai Cancer Center China; ^2^ Department of Oncology Shanghai Medical College Fudan University China; ^3^ Shanghai Medical College Collaborative Innovation Center of Cancer Medicine Fudan University Shanghai Cancer Center and Institutes of Biomedical Sciences Fudan University China

**Keywords:** colon cancer, early relapse, Gene Expression Omnibus database, mRNA signature, propensity score

## Abstract

Colon cancer patients experiencing early relapse consistently exhibited poor survival. The aim of our study was to develop an mRNA signature that can help to detect early relapse cases in stage I–III colon cancer. Public microarray datasets of stage I–III colon cancer samples were extracted from the Gene Expression Omnibus database. Propensity score matching analysis was performed between patients in the early relapse group and the long‐term survival group from GSE39582 discovery series (*N* = 386), and patients were 1 : 1 matched. Global mRNA expression changes were then analyzed between the paired groups to identify the differentially expressed genes. Lasso Cox regression modeling analysis was conducted for the selection of prognostic mRNA. Fifteen mRNA were finally identified to build an early relapse classifier. With specific risk score formula, patients were classified into a high‐risk group and a low‐risk group. Relapse‐free survival was significantly different between the two groups in every series, including discovery [hazard ratio (HR): 2.547, 95% confidence interval (CI): 1.708–3.797, *P* < 0.001)], internal validation (HR: 5.146, 95% CI: 1.968–13.457, *P* < 0.001), and external validation (HR: 1.977, 95% CI: 1.295–3.021, *P* < 0.001) sets of patients. Time‐dependent receiver‐operating characteristic at 1 year suggested more prognostic accuracy of the classifier [area under curve (AUC = 0.703)] than the American Joint Commission on Cancer tumor–node–metastasis staging system (AUC = 0.659) in all 951 patients. In conclusion, we developed a robust mRNA signature that can effectively classify colon cancer patients into groups with low and high risks of early relapse. This mRNA signature may help select high‐risk colon cancer patients who require more aggressive therapeutic intervention.

**Abbreviations**
AJCC: American Joint Commission on Cancer
ARP3: actin‐related protein 3 homolog B
AUC: area under curve
BLMH: bleomycin hydrolase
CCL20: C‐C motif chemokine ligand 20
CI: confidence interval
CMPK2: cytidine/uridine monophosphate kinase 2
CRC: colorectal cancer
CTCs: circulating tumor cells
DEGs: differentially expressed genes
ECM1: extracellular matrix protein 1
GEO: Gene Expression Omnibus
gPCA: guided PCA
GZMB: granzyme B
HES6: hes family BHLH transcription factor 6
HR: hazard ratio
IL7: interleukin 7
KLK10: kallikrein‐related peptidase 10
KRT6A: keratin 6A
LIMMA: linear models for microarray data
MMP9: matrix metallopeptidase 9
MSLN: mesothelin
OAS1: 2′‐5′‐oligoadenylate synthetase 1
PS: propensity score
PUS7: pseudouridylate synthase 7
ROC: receiver‐operating characteristic
TNM: tumor–node–metastasis
UICC: International Union against Cancer
ZNF426: zinc finger protein 426

## 1 Introduction

Colorectal cancer (CRC) is a worldwide common malignant tumor and also a major cause of cancer‐related deaths (Siegel *et al*., 2016). The implement of curative resection and advancement of adjuvant chemotherapy have witnessed a moderate improvement on the overall prognosis of colon cancer (Shi *et al*., 2013; Wilkinson *et al*., 2010). However, relapse following surgery is still a major and ultimate cause of deaths. Approximately 25–40% of patients would develop recurrence or metastases after primary radical resection, of which one‐third were local recurrence and the others were distal metastases (Becker, 1995; Tsai *et al*., 2009b; Van Cutsem *et al*., 2006). For the most part, the relapse of colon cancer is time‐related. Almost 40–50% of relapse emerged within the first year after initial primary resection, and 90% within the first 3 years (Longo and Johnson, 2002; Seo *et al*., 2013). Early relapse in colon cancer patients is attributed mainly to poor clinicopathological features (such as deeper tumor invasion, regional lymph nodes metastasis, poor differentiation, and worse histopathological type) and poor response to adjuvant chemotherapy. Those cases who developed early relapse consistently tended to have poorer long‐term survival rates. Consequently, more valuable predictive factors are urgently needed to detect the early postoperative relapse.

For decades, the most significant risk factor for predicting early relapse in colon cancer is based on American Joint Commission on Cancer/International Union against Cancer (AJCC/UICC) tumor–node–metastasis (TNM) staging system. However, for the great heterogeneity of colon cancer, prognosis varies significantly in colon cancer patients with same tumor stage and comparable clinicopathological features. Therefore, researchers are searching extensively for the ideal biomarker or indicator for predicting early relapse in colon cancer patients (Hwang *et al*., 2011; Lu *et al*., 2011; Yang *et al*., 2012). Although substantial efforts have been made to explore gene‐based molecular biomarker to predict the early relapse, no molecular prognostic classifiers have been established. Recent studies in many malignancies, including colon cancer, suggested that multigene expression patterns or gene signature can make a good prediction of cancer prognosis (Catto *et al*., 2010; Chen *et al*., 2011; Lee *et al*., 2015b; Tan and Tan, 2011). But, few precious gene profiling has been applied to detect the early relapse‐associated genes in colon cancer. Searching a gene signature might be of concrete predictive value in the prediction of early relapse in colon cancer patients.

In this study, a large group of mRNA‐specific probes were fortuitously represented on the commonly used microarray platform (Affymetrix HG‐U133 plus 2.0). We adopted previously published gene expression microarray data from the Gene Expression Omnibus (GEO) and conducted mRNA profiling on large cohorts of colon cancer patients. Using the sample‐splitting method and Cox regression analysis, a prognostic 15‐mRNA signature was identified from the discovery set in GSE39582 and validated in the internal validation series and another four GEO cohorts. This mRNA signature may help identify the subset of colon cancer patients at high risk of early relapse. Extensive postoperative management and surveillance may be needed for these patients.

## 2 Materials and methods

### 2.1 Preprocessing of microarray data

Raw microarray colon cancer datasets were obtained from the GEO database (http://www.ncbi.nlm.nih.gov/geo/) and were normalized using Robust Multichip Average (Irizarry *et al*., 2003). All datasets were produced by the Affymetrix HG‐U133 plus 2.0 platform. All probes were mapped based on their own EntrezGeneID. When multiple probes were mapped to the same EntrezGeneID, the mean value was used to represent its average expression level.

### 2.2 Dataset selection

The selection criterion for CRC datasets were as follows: (a) All sets were created by Affymetrix HG‐U133 plus 2.0 platform; (b) all sets should have basic clinical information of stage, relapse‐free survival (RFS) interval and RFS status; (c) datasets with larger sample size were preferred to be chosen, and we limited sample size to ~ 100. Datasets missing necessary clinicopathological or follow‐up data were excluded. Finally, CRC datasets of GSE39582, GSE14333, GSE17538, GSE33113, and GSE37892 were identified in this study to construct and validate the prognostic value of gene signature. Table S1 was used to describe the GEO datasets that were excluded in our study. All the samples were further filtered based on the criterion of stage I–III colon cancer and the availability of clinical outcome data. GSE39582 is the largest set consisting of 497 stage I–III colon cancer, and hence, it was assigned to a discovery series and an internal validation series. GSE17538, GSE14333, GSE33113, and GSE37892 were combined and set as external validation series. Analyses of the probe cell intensity (CEL) files suggested that there was extensive overlap between samples (H. Lee Moffitt Cancer Center) in the GSE17538 and GSE14333 series (Sveen *et al*., 2012). Therefore, the samples from Moffitt Cancer Center (*N* = 138, stages I–III) in GSE17538 were excluded. ComBat method was used to remove the internal batch effects among 10 batches in GSE39582 and external batch effects among different GEO datasets. This method was implemented in the SVA R package, and the corresponding R‐code can be found as Data S1. The guided PCA (gPCA) method was used to evaluate the success of batch effect correction (Reese *et al*., 2013).

### 2.3 Identification of early relapse‐associated genes

Early relapse was defined as the locoregional recurrence or distant metastasis within 1 year after primary resection (Lu *et al*., 2011). Samples in the discovery set from GSE39582 were selected and divided into early relapse group and long‐term survival group (no relapse after a minimum of 5 years follow‐up). Propensity score (PS) matching analysis was performed between the two groups to adjust for stage and adjuvant chemotherapy, which were the most significant clinical factors associated with early relapse. All patients were matched 1 : 1. Finally, 45 paired patients in the discovery set were identified to identify the changes of global gene expression profile between early relapse group and long‐term survival groups. The analysis of differentially expressed genes (DEGs) between early relapse and long‐term survival samples was conducted using the linear models for microarray data (LIMMA) method (Smyth, 2005). The threshold for identification of DEGs was set as *P* < 0.05 and fold change ≥ 1.25. Lastly, LASSO Cox regression model (Tibshirani, 1997) was used to select the most significantly relapse‐associated mRNA of all the DEGs.

### 2.4 Development of risk score and statistical analysis

Using LASSO Cox regression analysis, we identified a panel of genes and constructed a multi‐mRNA‐based classifier for predicting the early relapse in patients with stage I–III colon cancer in the discovery set. With specific risk score formula, patients from different sets were divided into high‐risk and low‐risk groups using the median risk score of the discovery set as the cutoff point. Survival differences between the low‐risk and high‐risk groups in each set were assessed by the Kaplan–Meier estimate and compared using the log‐rank test. Multivariate Cox regression analysis and data stratification analysis were performed to test the independent prognostic role of risk score in predicting RFS. Time‐dependent receiver‐operating characteristic (ROC) analysis was used to investigate the prognostic or predictive accuracy of each feature and signature. All statistical analyses were performed with use of r (version 2.15.0, http://www.r-project.org). All statistical tests were two‐sided, and *P* values < 0.05 were considered statistically significant.

## 3 Results

### 3.1 Preparation of colon cancer datasets

A total of 951 patients were identified and fully studied, which included 497 patients from GSE39582 (386 patients from the discovery set and 111 from the internal validation set), 234 patients from GSE17538 and GSE14333, 90 patients from GSE33113, and 130 patients from GSE37892. Plots of the first versus the second principal components before and after removing batch effects are shown in Fig. S1. The original data of the all patients included in analysis are listed in Table S2. The baseline clinical information for patients in external validation sets, GSE14333, GSE17538, GSE33113, and GSE37892, is shown in Table S3.

### 3.2 Development of early relapse signature from the discovery series

**Figure 1:**
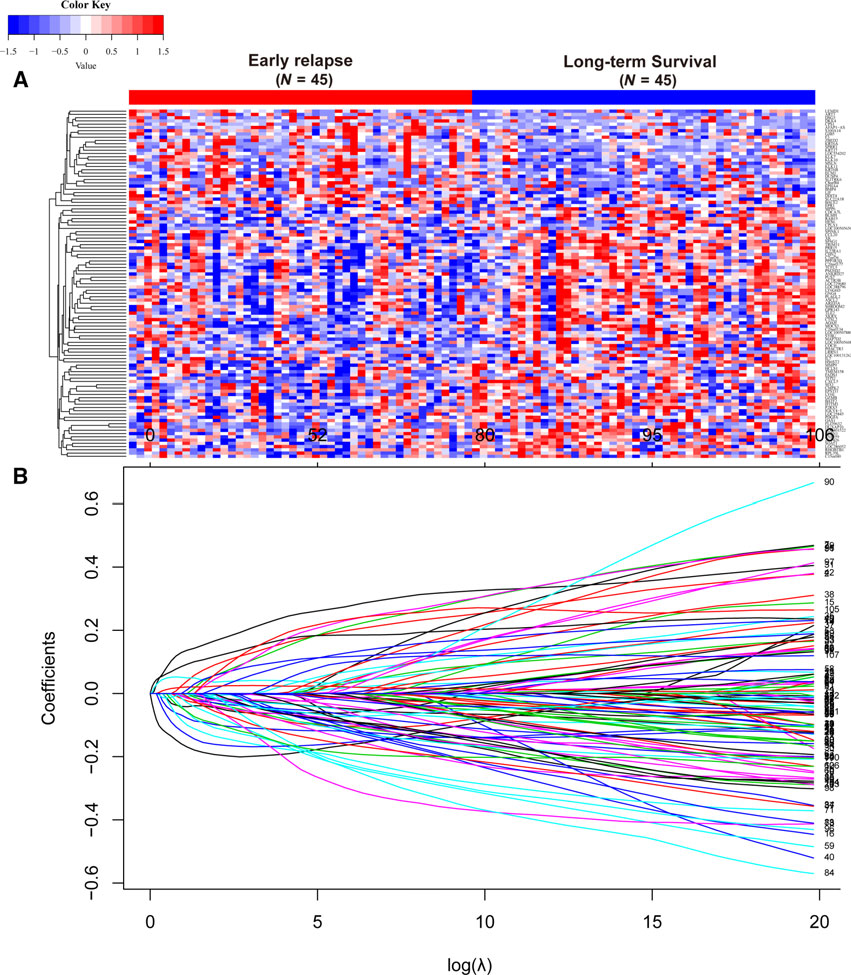
(A) Heat map showed eighteen differentially expressed mRNA in colon cancer between early relapse and long‐term survival group both in discovery set. (B) LASSO coefficient profiles of the 107 early relapse‐associated mRNA. A vertical line is drawn at the value chosen by 10‐fold cross‐validation.

Samples in discovery set were divided into early relapse group and long‐term survival group. Patients' clinicopathological features before and after PS matching are described in Table 1. Before the implement of PS analysis, it is noticeable that tumor stage in early relapse group was significantly higher than that in long‐term survival group. After PS matching, there were no significant differences in age, AJCC stage, tumor location, and adjuvant chemotherapy between early relapse and long‐term survival groups in each set (Table 1). Changes of global mRNA expression profiles were analyzed between early relapse and long‐term survival groups. One hundred and seven of them were differentially expressed between the two groups (*P* < 0.05, fold change ≥ 1.25; Fig. 1A). LASSO coefficient profiles of the 107 mRNA are shown in Fig. 1B. A coefficient profile plot was produced against the log (λ) sequence. Vertical line was drawn at the value selected using 10‐fold cross‐validation, and the minimize λ method resulted in 15 optimal coefficients. Of these, 11 mRNA were downregulated and four were upregulated in early relapse group compared with long‐term survival group (Table S4). Using Lasso Cox regression modeling, we derived a 15‐mRNA signature to calculate the risk score for every patient based on the expression levels of the 15 RNA weighted by their regression coefficients: risk score = (− 0.052 × expression level of *ACTR3B*) + (− 0.116 × expression level of *BLMH*) + (− 0.047 × expression level of *CCL20*) + (− 0.121 × expression level of *CMPK2*) + (0.259 × expression level of *ECM1*) + (0.043 × expression level of *GZMB*) + (− 0.287 × expression level of *HES6*) + (− 0.102 × expression level of *IL7*) + (0.201 × expression level of *KLK10*) + (− 0.015 × expression level of *KRT6A*) + (− 0.302 × expression level of *MMP9*) + (0.038 × expression level of *MSLN*) + (− 0.217 × expression level of *OAS1*) + (− 0.236 × expression level of *PUS7*) + (− 0.168 × expression level of *ZNF426*).

**Table 1:** Clinical–pathological features of patients in early relapse and long‐term survival groups before and after PS matching

Variable	Discovery set					
	Before matching			After matching		
	Early relapse	Long‐term survival	*P*	Early relapse	Long‐term survival	*P*
Age (mean, IQR)	68.2 (58.5–78.0)	65.2 (57.5–73)	0.17	68.2 (58.0–78.0)	66.4 (60.0–73.0)	0.52
Gender						
Male	20	66	0.75	20	19	0.83
Female	25	74		25	26	
Stage						
I	0	8	0.08	0	0	1
II	20	76		20	20	
III	25	56		25	25	
T stage						
T1	0	3	0.03	0	1	0.16
T2	2	11		2	3	
T3	25	108		25	35	
T4	13	18		13	6	
NA	5	0		5	0	
N stage						
N0	24	84	0.01	16	20	0.09
N1	11	39		11	19	
N2	13	17		13	6	
NA	1	0		1	0	
Tumor location						
Proximal	17	54	0.92	17	19	0.67
Distal	28	86		28	26	
Adjuvant chemotherapy						
No	20	80	0.14	20	20	1
Yes	25	60		25	25	
Total	45	140		45	45	

### 3.3 The prognostic value of 15‐mRNA signature in discovery, internal validation, and external validation series

**Figure 2:**
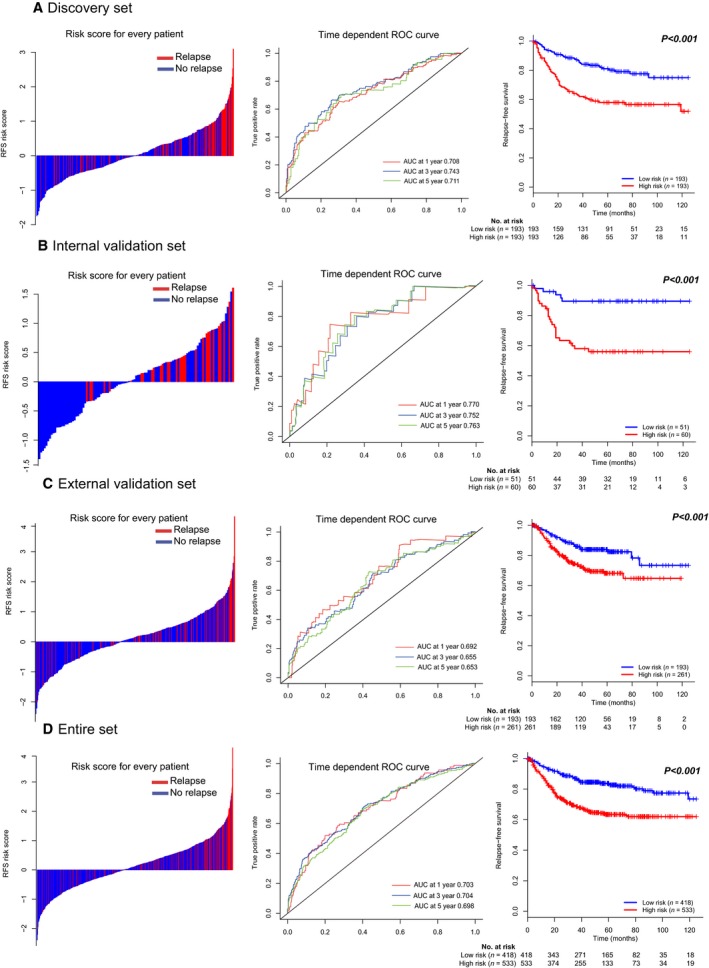
Distribution of risk score, time‐dependent ROC curves at 1, 3, and 5 years and Kaplan–Meier survival analysis between patients at low and high risks of relapse in discovery set (A), internal validation set (B), external validation set (C), and entire dataset (D).

Patients in discovery set were divided into low‐risk group (*N* = 193) or high‐risk group (*N* = 193) using the median risk score as cutoff point. The distribution of risk scores and survival status is shown in Fig. 2A (left panel), which suggested that patients with lower risk scores generally had better survival than those with higher risk scores. Time‐dependent ROC analyses at 1, 3, and 5 years were conducted to assess the prognostic accuracy of the 15‐mRNA‐based classifier (Fig. 2A, middle panel). The RFS rates for patients with low‐risk scores were 93.6% at 1 year, 86.6% at 3 years, and 81.0% at 5 years, compared with 83.4%, 62.7%, and 57.9% in patients with high‐risk scores, respectively [hazard ratio (HR): 2.547, 95% confidence interval (CI): 1.708–3.797, *P* < 0.001, Fig. 2A, right panel].

We then did the same analyses in the internal validation cohort. In the internal validation series, 1‐, 3‐, and 5‐year RFS was 84.6%, 58.0%, and 56.0% for the high‐risk group, and 95.9%, 89.5%, and 89.5% for the low‐risk group (HR: 5.146, 95% CI: 1.968–13.457, *P* < 0.001, Fig. 2B).

To confirm that the 15‐mRNA‐based classifier had similar prognostic value in different populations, we combined the samples in GSE17538, GSE14333, and GSE33113, and a total of 324 colon cancer patients were further studied to validate the 15‐mRNA signature. Using the established cutoff point, 193 (42.5%) patients were classified as low risk, and 261 (57.5%) as high risk. Five‐year disease‐free survival was 68.1% for the high‐risk group and 83.9% for the low‐risk group (HR: 1.977, 95% CI: 1.295–3.021; *P* < 0.001; Fig. 2C).

In the entire dataset analysis, risk score‐based classification yielded similar results (Fig. 2C). Colon cancer patients can be divided into low‐ and high‐risk patients with significantly different RFS, and the signature showed the highest predicting accuracy at 1 year after surgery.

### 3.4 Independence and accuracy of the signature in predicting RFS

**Figure 3:**
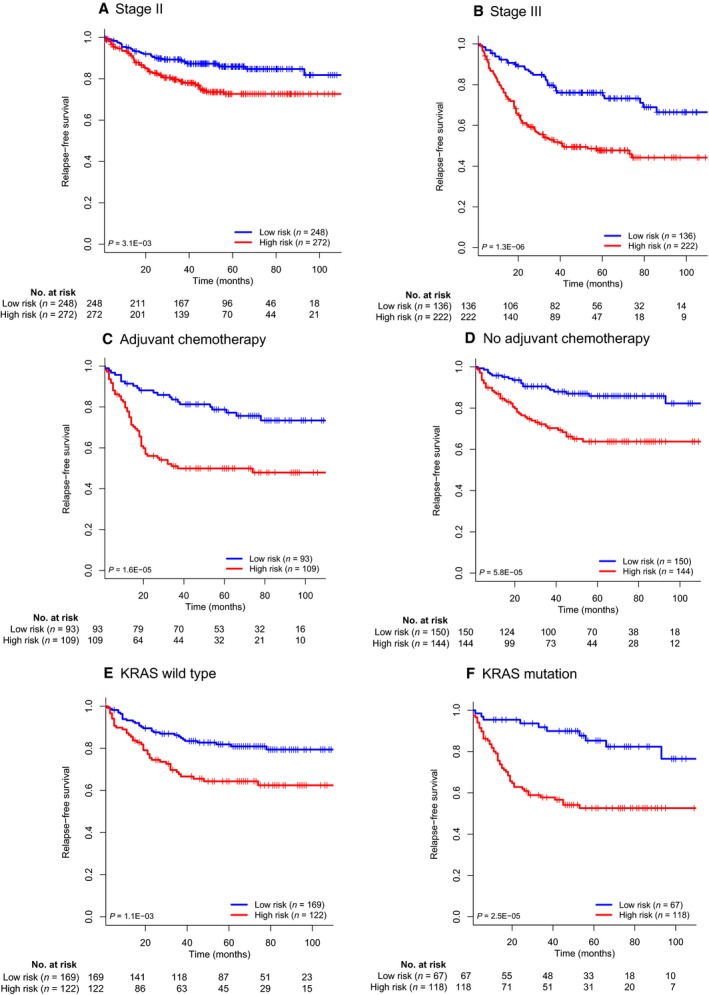
Kaplan–Meier survival analysis for the entire dataset with stage I–III colon cancer (*N* = 951) based on the 15‐mRNA‐based signature stratified by clinicopathological risk factors.

After multivariate analysis adjusted by clinicopathological variables, the 15‐mRNA‐based classifier remained a powerful and independent factor in the discovery, internal validation, and external validation sets (Table 2). Stratified analysis suggested that the 15‐mRNA‐based classifier was still a clinically and statistically significant prognostic model in stage II, stage III, patients with or without adjuvant chemotherapy and patients with or without *KRAS* mutation (Fig. 3). Samples from the entire dataset were then separated into five risk groups based on their relapse‐free status and time: group A (relapse within 1 year), group B (relapse within 3 years), group C (relapse after 3 years), group D (no relapse within 5 years), group E (no relapse after minimum 5 years). The distribution of risk score among five risk groups are shown in Fig. S2. As expected, group A showed the highest risk score, while group E showed the lowest.

**Table 2:** Univariable and multivariable Cox regression analysis in colon cancer

Variables	Univariate analysis		Multivariate analysis	
	HR (95% CI)	*P*	HR (95% CI)	*P*
Discovery set (*N* = 386)				
Age	1.01 (0.99 to 1.02)	0.438	1.01 (0.99 to 1.03)	0.132
15 gene risk score	2.58 (2.04 to 3.28)	< 0.001	2.52 (1.97 to 3.23)	< 0.001
Gender				
Female	1	0.109	1	0.167
Male	1.36 (0.93 to 2.01)		1.31 (0.99 to 1.02)	
Stage				
I	1	0.005	1	0.161
II	6.44 (0.88 to 46.67)		5.45 (0.75 to 39.70)	
III	10.43 (1.44 to 75.29)		6.67 (0.89 to 49.98)	
Tumor location				
Proximal	1	0.52	1	0.179
Distal	1.13 (0.77 to 1.66)		0.76 (0.51 to 1.15)	
Adjuvant chemotherapy				
No	1	0.001	1	0.296
Yes	1.85 (1.27 to 2.70)		1.30 (0.79 to 2.14)	
Internal validation set (*N* = 111)				
Age	1.02 (0.98 to 1.05)	0.221	1.01 (0.97 to 1.05)	0.324
15 gene risk score	3.57 (1.97 to 6.45)	< 0.001	2.86 (1.51 to 5.40)	< 0.001
Gender				
Female	1	0.442	1	0.612
Male	1.34 (0.63 to 2.87)		1.24 (0.53 to 2.87)	
Stage				
I	1	0.003	1	0.023
II	> 1000 (0 to > 1000)		> 1000 (0 to > 1000)	
III	> 1000 (0 to > 1000)		> 1000 (0 to > 1000)	
Tumor location				
Proximal	1	0.798	1	0.367
Distal	1.10 (0.51 to 2.36)		0.69 (0.30 to 1.56)	
Adjuvant chemotherapy				
No	1	0.237	1	0.257
Yes	1.54 (0.75 to 3.15)		0.56 (0.20 to 1.53)	
External validation set (*N* = 454)				
Age	0.99 (0.97 to 1.00)	0.116	0.99 (0.98 to 1.01)	0.863
15 gene risk score	1.79 (1.45 to 2.21)	< 0.001	1.60 (1.29 to 1.98)	< 0.001
Gender				
Female	1	0.798	1	0.972
Male	1.05 (0.71 to 1.55)		1.01 (0.67 to 1.50)	
Stage				
I	1	< 0.001	1	< 0.001
II	5.72 (0.78 to 41.7)		5.20 (1.25 to 21.64)	
III	18.95 (2.62 to 136.6)		11.94 (2.90 to 49.13)	

**Figure 4:**
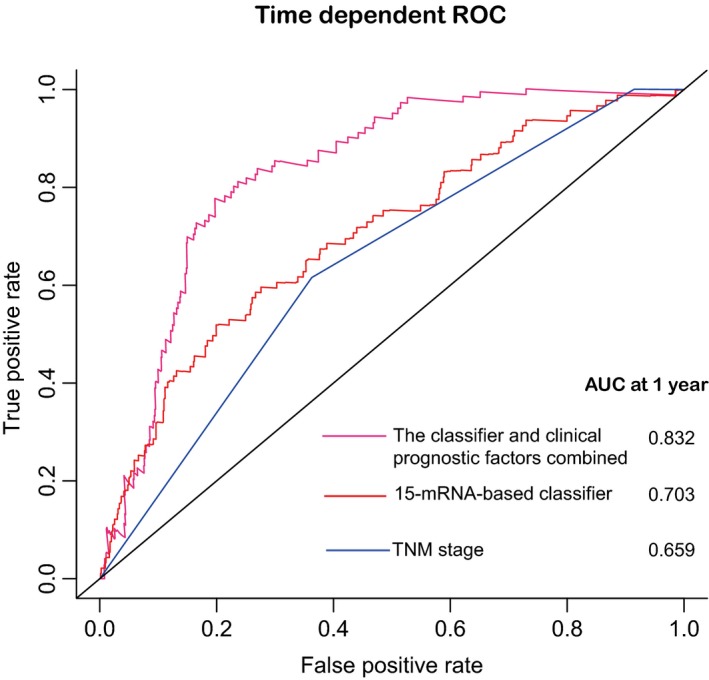
Time‐dependent ROC curves at 1 year compare the prognostic accuracy in predicting early relapse of the 15‐mRNA signature with TNM staging system in the entire cohorts with stage I–III colon cancer (*N* = 951).

To confirm that the 15‐mRNA‐based classifier had higher efficacy in predicting early relapse, time‐dependent ROC was used, which suggested that the 15‐mRNA‐based classifier had significantly higher prognostic accuracy than tumor stage at 1 year (Fig. 4).

### 3.5 Identification of 15‐mRNA signature‐associated biological signaling pathway

We performed GSEA in dataset GSE39582 to identify the 15‐mRNA‐associated biological signaling pathway. Significant gene sets (FDR < 5%) were visualized as Enrichment Map (Fig. S3). The risk score was accompanied with exceptional regulation of several important cancer‐related networks, namely apical junction, hypoxia, Hedgehog signaling, epithelial–mesenchymal transition, G2M checkpoint, interferon GAMMA response.

## 4 Discussion

To be noted, tumor relapse within the first year after initial resection still accounted for almost half of all tumor relapse, indicating that potential micrometastases, incomplete clinicopathological factors, or inherent heterogeneity may be critical factors in promoting tumor recurrence or distant metastasis (Steinert *et al*., 2008; Zhang *et al*., 2005). Postoperative relapse after radical surgery, ascribed to tumor cell dissemination, is closely related to survival outcomes, which is mainly evaluated by current AJCC/UICC TNM staging system. However, colon cancer patients within the same pathologic staging vary considerably in disease progression and prognosis due to their different genetic and epigenetic backgrounds, resulting in the unsatisfactory situation of current methods (Bathe and Farshidfar, 2014). Despite the continuous improvement of treatment strategies, patients with early postoperative relapse in colon cancer suffer from significantly inferior overall survival rates, in comparison with those without early relapse (Hwang *et al*., 2011). Simple and reliable biomarkers for the detection of early postoperative relapse would make up for the deficiency of standard TNM classification, and thereby assisting physicians in formulating more efficient therapeutic strategies at an earlier stage of a patient's treatment (Cho, 2010; Tsai *et al*., 2013a).

Previous studies have tried to identify postoperative molecular markers for detection of early relapse in colon cancer. In 2009, Tsai *et al*. (Tsai *et al*., 2009a) indicated that vascular invasion, perineural invasion, and postoperative CEA level may be significant factors for postoperative early relapse in UICC stage I–III colon cancer. Besides, it is also demonstrated in 2013 that activation of VEGF, an important predictor of early postoperative relapse in patients with stage I–III CRC, may help identify patients who would benefit from intensive follow‐up and therapeutic programs (Tsai *et al*., 2013b). Recently, another research conducted by Lu *et al*. (2011) revealed that molecular detection of persistent postoperative circulating tumor cells (CTCs) is a prognostic predictor of early relapse in UICC stage II/III colon cancer patients. Nonetheless, although the association between molecular markers and early postoperative relapse in colon cancer has been explored in relevant literatures, most work has focused on analyzing the function of one or two biomarkers. Little attention has been paid to mRNA expression pattern and its involvement in the prediction of early relapse in stage I–III colon cancer using high‐throughput expression profile datasets.

In the current study, a novel prognostic classifier based on 15 mRNA was developed to improve the prediction of early relapse and prediction of RFS for colon cancer after surgical resection. By applying the 15‐mRNA signature to the GSE39582 discovery set patients, a clear separation was observed in the survival curves between low‐ and high‐risk patients. And it was internally validated in the validation series of GSE39582 patients and the external cohorts of GSE17538, GSE14333, GSE33113, and GSE37892, indicating the good reproducibility of this signature in colon cancer. Stratified by AJCC stage, the 15‐mRNA‐based signature remains a good prognostic model, implying that the mRNA signature can be used to refine the current staging system. Furthermore, the time‐dependent ROC at 1 year suggested that this 15‐mRNA signature has considerable prognostic accuracy in predicting tumor relapse within the first year after initial resection of colon cancer. Therefore, our study identified a 15‐mRNA signature that could help identify patients with high risk of early relapse and guide individualized treatment of patients with colon cancer, which is credible to be applied to clinic.

Most of genes included in the signature have been experimentally demonstrated to be linked with cancer. Of these, six mRNA including *ECM1, GZMB, KLK10, CCL20, MMP9*,* and IL7* have been previously reported to have a prognostic role in colon cancer. Extracellular matrix protein 1 (ECM1) is a secreted protein that has been implicated with cell proliferation, angiogenesis, and differentiation (Lee *et al*., 2015a). Previous studies suggested that ECM1 tends to be preferentially expressed by metastatic CRC (Wang *et al*., 2003). Granzyme B (GZMB) is a serine protease expressed by cytotoxic T lymphocytes and natural killer cells (Dahl *et al*., 1990). Patients with low expression of GZMB have been proved to have poor disease‐free survival (Tosolini *et al*., 2011). Kallikrein‐related peptidase 10 (KLK10) is homologue to KLK3 and encodes the prostate‐specific antigen, which is a widely used biomarker for the detection and monitoring of prostate cancer (Sardana *et al*., 2007). The mRNA expression level of *KLK10* has been previously suggested to be negatively associated with prognosis in CRC (Alexopoulou *et al*., 2013). Several recent studies suggested that CC‐chemokine cysteine motif chemokine ligand 20 (CCL20) and its physiological sole receptor CCR6 played a role in the development and metastatic spread of CRC (Ghadjar *et al*., 2009; Iwata *et al*., 2013). However, this hypothesis was warranted to be further validated by functional studies and the results from Ghadjar *et al*. (2006) did not support it. Matrix metalloproteinase (MMP) played an important role in degradation of extracellular matrix and basement membranes, and previous studies indicated that the overexpression of matrix metallopeptidase 9 (MMP9) was associated with deep tumor invasion, lymph‐node metastasis, and advanced TNM stage in CRC (Lee *et al*., 2014; Matsuyama *et al*., 2002). But a recent study revealed that overexpression of MMP9 can predict good response to chemotherapy in patients with CRC (Yang *et al*., 2017). Therefore, we hypothesized that in patients with comparable clinicopathological features, those with high expression level of MMP9 may exhibit better survival, which has been suggested by the results in this study. Interleukin‐7 (IL‐7) is a cytokine that has been known since long in immunology, and recent studies found the role of IL‐7 was far beyond the field of immunology and it might have direct or indirect effect on cancer (Lin *et al*., 2017). However, its prognostic and biological effects varied significantly among different studies and cancer types (Berghella *et al*., 2002; Fritzell *et al*., 2013; Liu *et al*., 2014; Lynch *et al*., 1991). In our study, we found the expression of *IL7* was upregulated in long‐term survival group and may exert antitumor effect. Bleomycin hydrolase (BLMH) is a kind of drug‐metabolizing enzymes that were highly expressed in drug‐resistant colon cancer stem cells, but no previous studies was conducted to detect the prognostic role of BLMH in colon cancer (Emmink *et al*., 2013). As for the rest eight genes integrated in our signature, further clinical and basic research should be conducted to explore their value in colon cancer.

To date, several multigene assays have been developed like Oncotype DX (Webber *et al*., 2010), ColoPrint (Salazar *et al*., 2011) and ColDX (Kennedy *et al*., 2011), in hopes of providing prognostic and predictive information to aid in decisions regarding adjuvant therapy in patients with stage II or III colon cancer. However, before the signature can be applied as a clinical‐grade assay, further steps are needed according to the established guidelines (Altman *et al*., 2012): firstly, identification of an appropriate approach to quantify expression (microarray); secondly, design of specific probes based on the sequences tested in the microarray chips; thirdly, validation in independent cohorts of patients with full clinical annotation available. We will firstly validate the prognostic value of this classifier in our center. Although not controversially applicable worldwide in the present form, we do believe the multigene classifier established in this study bears promising translational value.

Inevitably, there are some limitations in our study. Firstly, our study was based on the data from a publicly available datasets without testing prospectively in a clinical trial. Furthermore, the information of several other important clinicopathological features, like differentiation and number of lymph nodes, was not available in these datasets. Finally, mechanisms of the identified genes on the early relapse in colon cancer are still needed to be further explored.

## 5 Conclusions

In conclusion, we developed a robust mRNA signature consisting of both up‐ and downregulated mRNA that can effectively classify colon cancer patients into groups with low and high risks of early relapse postoperatively. Further validation in prospective clinical trials could verify the clinical significance of this mRNA signature in detecting postoperative early relapse in colon cancer patients.

## Author contributions

WXD and GXC had the idea for this study. QGL supervised the acquisition of the data. YF undertook the statistical analysis, and YX and LZ provided statistical advice. All authors contributed to interpretation of the results. WXD, YQL, and SBM wrote the article, and other authors contributed to the content. All authors approved the final version of the manuscript, including the authorship list.

## Supporting information


**Fig. S1**. Principal components plot of first two principal components from gPCA.

**Fig. S2**. The distribution of risk score among five risk groups in the entire dataset with stage I–III colon cancer (*N* = 951).

**Fig. S3**. Gene set enrichment analysis delineates biological pathways associated with risk score.

**Table S1**. Description of GEO datasets that were excluded in our study.

**Table S2**. Clinicopathological features of patients in GSE39582, GSE1433, GSE33113, GSE17538 and GSE37892.

**Table S3**. Baseline information for patients in GSE14333, GSE33113, GSE17538 and GSE37892.

**Table S4**. Detailed information of eighteen gene identified from discovery series in GSE39582

**Data S1**. R code for batch effect removing.
