# Supplementary figures and images for: A robust gene signature for the prediction of early relapse in stage I–III colon cancer
Source: Mol Oncol. 2018 Feb 16;12(4):463–75. doi: 10.1002/1878-0261.12175 (PMC5891048; doi:10.1002/1878-0261.12175)

Figure S1

A. Internal batch effects

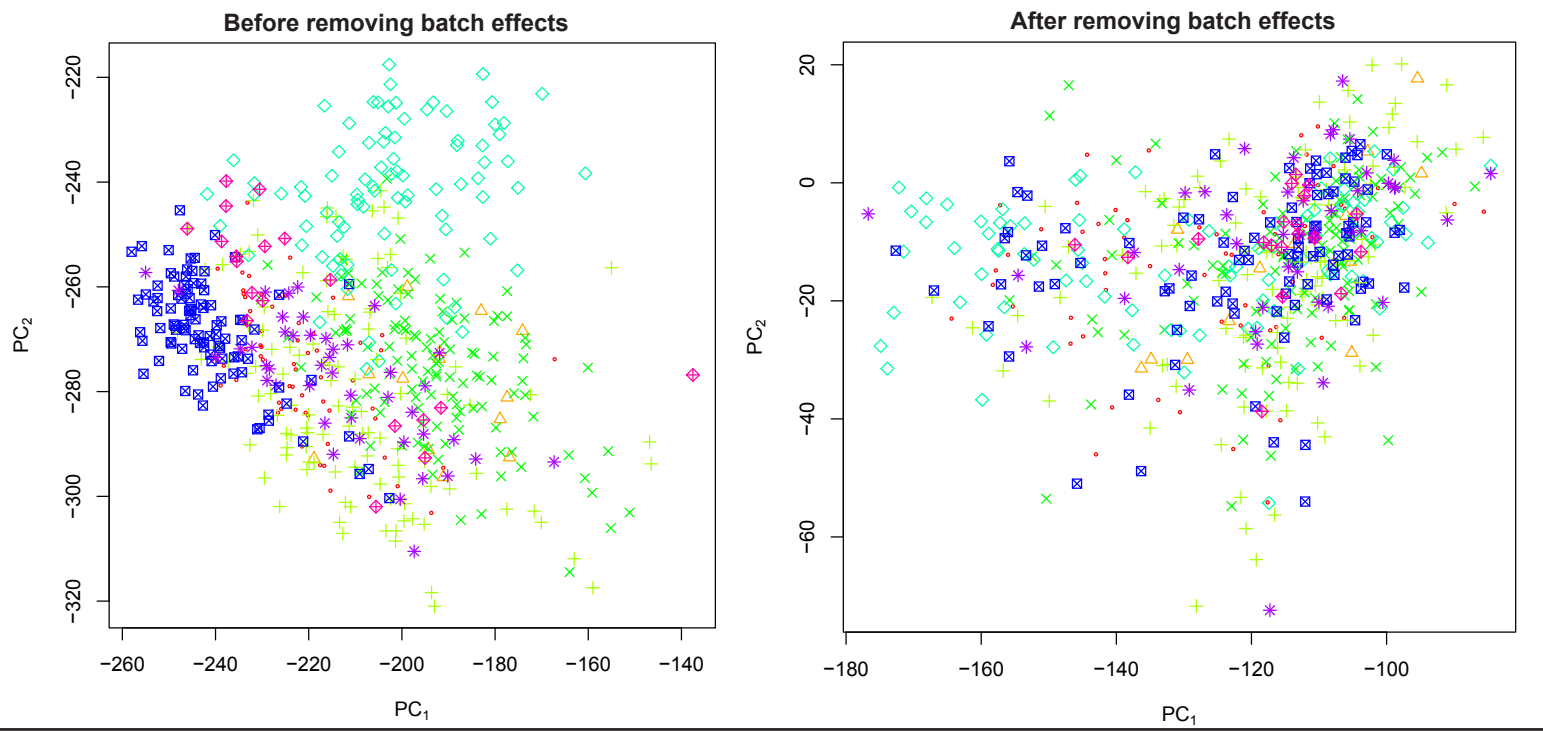

B. Overall batch effects

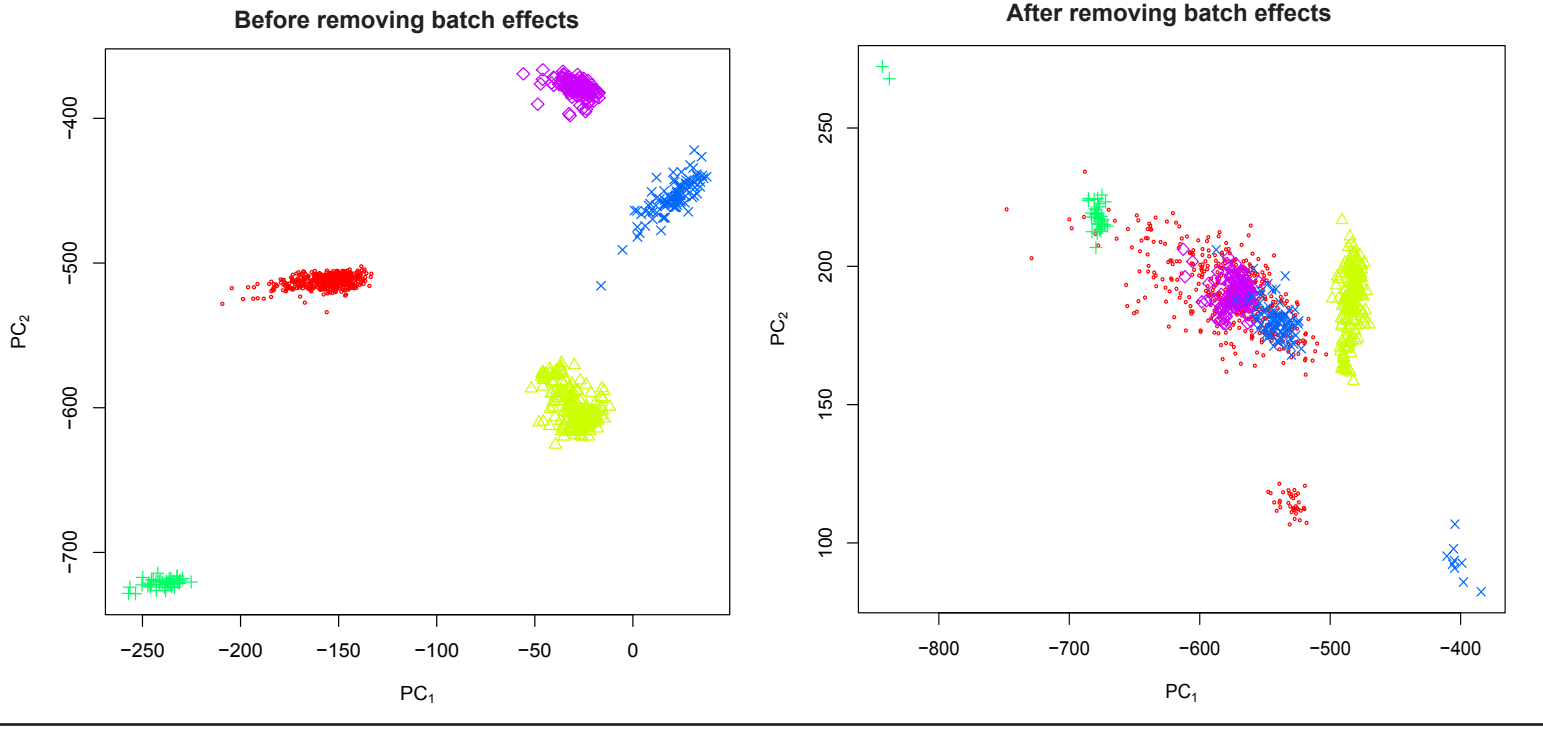

Supplement: Supplementary file 1 — Fig. S1. Principal components plot of first two principal components from gPCA. [file MOL2-12-463-s001.pdf]

**Figure S2**

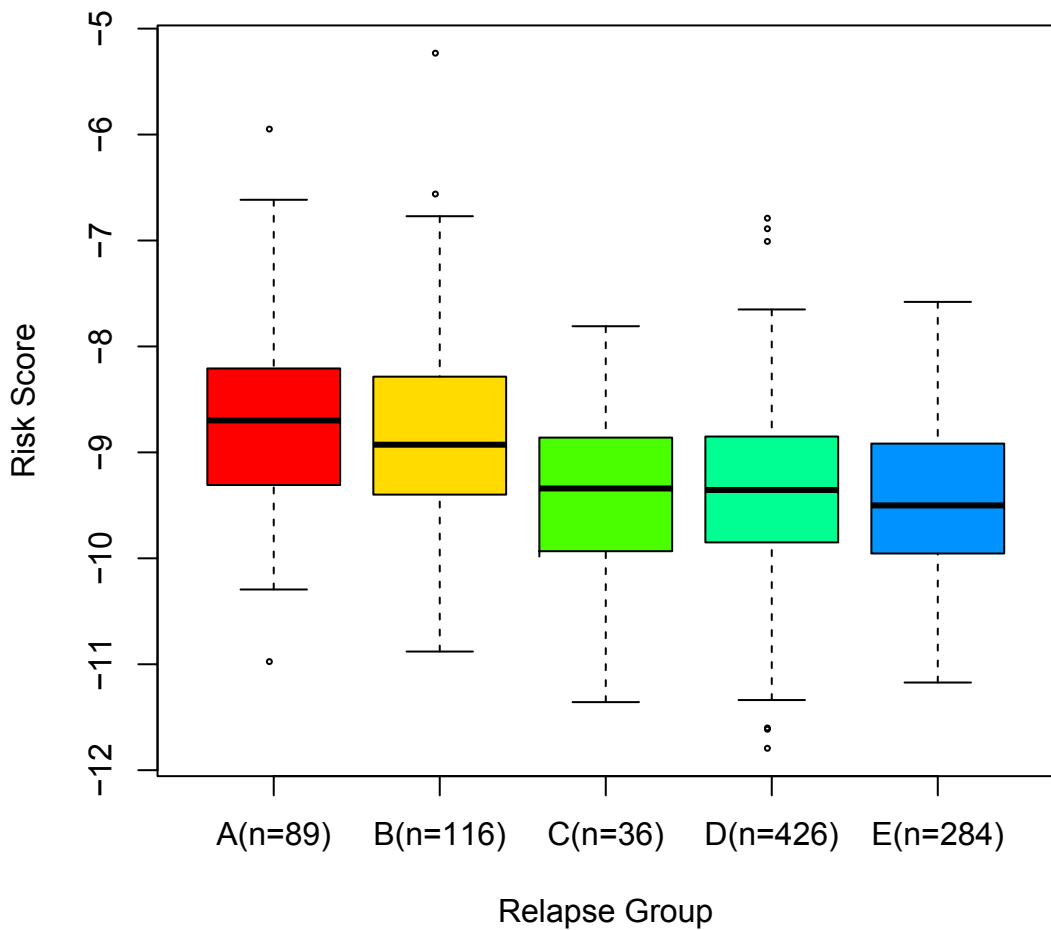

Supplement: Supplementary file 2 — Fig. S2. The distribution of risk score among five risk groups in the entire dataset with stage I–III colon cancer (N = 951). [file MOL2-12-463-s002.pdf]

Figure S3

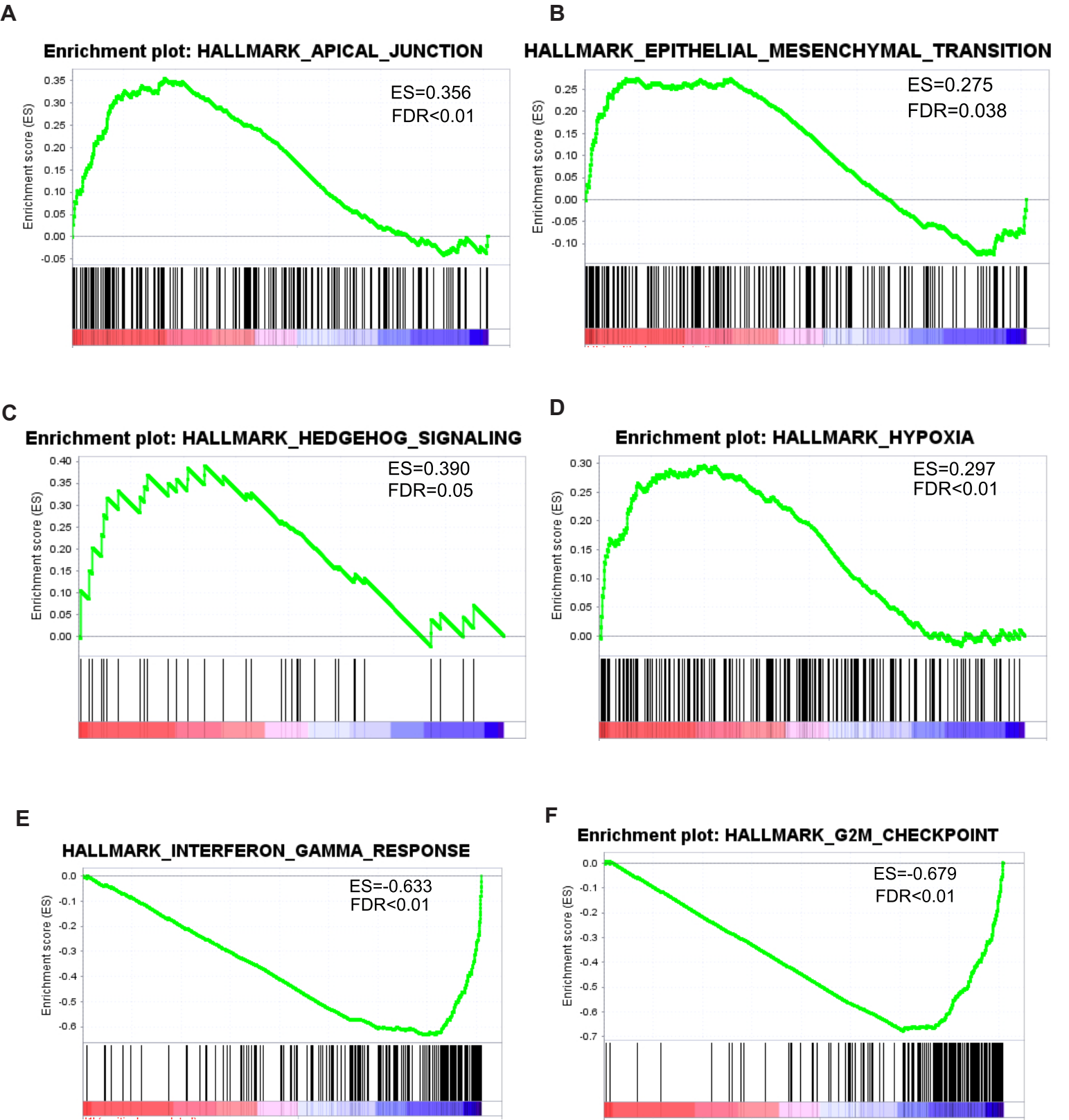

Supplement: Supplementary file 3 — Fig. S3. Gene set enrichment analysis delineates biological pathways associated with risk score. [file MOL2-12-463-s003.pdf]
