# Supplementary material for: A robust gene signature for the prediction of early relapse in stage I–III colon cancer
Source: Mol Oncol. 2018 Feb 16;12(4):463–75. doi: 10.1002/1878-0261.12175 (PMC5891048; doi:10.1002/1878-0261.12175)
Supplement: Supplementary file 4 — Table S1. Description of GEO datasets that were excluded in our study. [file MOL2-12-463-s004.docx]

| **Table S1. Description of GEO datasets that were excluded in our study.** | | |
| --- | --- | --- |
| GEO series | number of colon cacner patients | Reason to be excluded |
| GSE64256 | unknown | No basic clinical information |
| GSE27854 | unknown | No basic clinical information |
| GSE75315 | unknown | No basic clinical information |
| GSE71222 | unknown | No basic clinical information |
| GSE51244 | unknown | No basic clinical information |
| GSE13294 | unknown | No basic clinical information |
| GSE52735 | unknown | No basic clinical information |
| GSE14095 | unknown | No basic clinical information |
| GSE41568 | unknown | No basic clinical information |
| GSE38832 | unknown | No basic clinical information |
| GSE18105 | unknown | No basic clinical information |
| GSE33114 | unknown | No basic clinical information |
| GSE4459 | unknown | No basic clinical information |
| GSE4454 | unknown | No basic clinical information |
| GSE26682 | 176 | No follow-up information |
| GSE20916 | 145 | No follow-up information |
| GSE37364 | 94 | No follow-up information |
| GSE20906 | 90 | No follow-up information |
| GSE72968 | unknown | No stage I-III colon cancer |
| GSE81986 | unknown | Not fresh frozen tissuse |
| GSE29621 | 65 | small sample size |
| Footnote:*All listed or not listed series were created by Affymetrix HG-U133 plus 2.0 platform.**Tested specimens are colorectal cancer tissues.***There another 176 series with smaller sample size were not listed in this table. | | |
|  |  |  |
