# Supplementary material for: A robust gene signature for the prediction of early relapse in stage I–III colon cancer
Source: Mol Oncol. 2018 Feb 16;12(4):463–75. doi: 10.1002/1878-0261.12175 (PMC5891048; doi:10.1002/1878-0261.12175)
Supplement: Supplementary file 6 — Table S3. Baseline information for patients in GSE14333, GSE33113, GSE17538 and GSE37892. [file MOL2-12-463-s006.docx]

**Table S3. Baseline information for patients in GSE14333, GSE33113, GSE17538 and GSE37892.**

|  | GSE14333+  GSE17538 | % | GSE33113 | % | GSE37892 | % |
| --- | --- | --- | --- | --- | --- | --- |
| Age(IQR) | 65.6(56-75) |  | 70.4(61-79) |  | 68.2(59-76) |  |
| Gender |  |  |  |  |  |  |
| Male | 122 | 52.1 | 42 | 46.7 | 61 | 46.9 |
| Female | 112 | 47.9 | 48 | 53.3 | 69 | 53.1 |
| Stage |  |  |  |  |  |  |
| I | 37 | 15.8 | 0 | 0 | 0 | 0 |
| II | 97 | 41.5 | 90 | 1 | 73 | 56.2 |
| III | 100 | 42.7 | 0 | 0 | 57 | 43.8 |
| Total | 196 | 1.00 | 90 | 1 | 176 | 1 |
