# Supplementary material for: A robust gene signature for the prediction of early relapse in stage I–III colon cancer
Source: Mol Oncol. 2018 Feb 16;12(4):463–75. doi: 10.1002/1878-0261.12175 (PMC5891048; doi:10.1002/1878-0261.12175)
Supplement: Supplementary file 7 — Table S4. Detailed information of eighteen gene identified from discovery series in GSE39582 [file MOL2-12-463-s007.docx]

**Table S4 Detailed information of eighteen gene identified from discovery series in GSE39582**

| Gene Symbol | Discovery Set | |
| --- | --- | --- |
|  | logFC | P.Value |
| Down-regulated | | |
| BLMH | 0.327399 | 0.002537 |
| PUS7 | 0.332476 | 0.027995 |
| ZNF426 | 0.335204 | 0.017562 |
| ACTR3B | 0.379586 | 0.001781 |
| OAS1 | 0.421175 | 0.009569 |
| CMPK2 | 0.423979 | 0.044624 |
| IL7 | 0.431943 | 0.035483 |
| HES6 | 0.443127 | 0.012659 |
| MMP9 | 0.60779 | 0.007108 |
| CCL20 | 0.7157 | 0.043765 |
| GZMB | 0.721627 | 0.011526 |
| Up-regulated | | |
| MSLN | -1.11483 | 0.000973 |
| KRT6A | -0.70808 | 0.012673 |
| KLK10 | -0.64954 | 0.006959 |
| ECM1 | -0.3275 | 0.039931 |
